# Adjuvant and Perioperative Systemic Therapy for Resectable Hepatocellular Carcinoma: Evidence, Biological Rationale, and Trial Design

**DOI:** 10.3390/cancers18193126

**Published:** 2026-09-27

**Authors:** Xin Xin, Ruoyu Miao

**Affiliations:** 1 BayCare Health Systems, St. Joseph’s Hospital, Tampa, FL 33607, USA; xin.xin@baycare.org; 2 Department of Hematology and Medical Oncology, Winship Cancer Institute, Emory University, Atlanta, GA 30322, USA

**Keywords:** hepatocellular carcinoma, immunotherapy, targeted therapy, antiangiogenic therapy, neoadjuvant therapy, adjuvant therapy, perioperative therapy, molecular residual disease, circulating tumor DNA, microvascular invasion

## Abstract

Recurrence after curative-intent resection or ablation remains a major barrier to cure in hepatocellular carcinoma (HCC). Postoperative sorafenib was neutral in STORM, the initial recurrence-free survival signal with atezolizumab plus bevacizumab in IMbrave050 was not sustained with longer follow-up, and pembrolizumab was neutral in KEYNOTE-937. By contrast, perioperative camrelizumab plus rivoceranib improved event-free survival in CARES-009. This focused narrative review separates direct HCC clinical evidence from preclinical, cross-tumor, and translational evidence and addresses three development questions: how to test the intact-tumor antigen-source hypothesis, how to select patients biologically, and how to advance perioperative regimens without compromising curative surgery. The primary tumor and its draining lymphatic network may support antigen presentation and expansion of tumor-reactive lymphocytes during checkpoint blockade, but no randomized HCC trial has isolated treatment timing while holding regimen, exposure, population, and time origin constant. CARES-009 therefore validates one complete perioperative strategy rather than proving that neoadjuvant exposure caused benefit. Biological selection should proceed across the treatment pathway: pretreatment clinical risk and liver reserve establish eligibility; on-treatment safety and tissue response assess pharmacodynamic activity; and postoperative pathology, circulating tumor DNA, alpha-fetoprotein/des-gamma-carboxy prothrombin kinetics, and spatial immune features refine residual risk. These markers are prognostic, but none has demonstrated treatment-predictive utility in a randomized biomarker-guided trial. A stage-gated development program should establish agent-specific dose and washout, preserve operability, separate timing and treatment components in randomized phase II studies, and then confirm durable event-free and overall survival in globally representative phase III trials. Durable cure without loss of surgical opportunity, hepatic reserve, or quality of life should define success.

## Simple Summary

Even after hepatocellular carcinoma, the most common primary liver cancer, is completely removed, it often returns. Treatments started only after surgery have not consistently produced durable benefits. In CARES-009, camrelizumab plus apatinib (rivoceranib; hereafter, rivoceranib) given before and after surgery delayed cancer-related events compared to surgery alone, but the trial did not show which part of that treatment plan caused the benefit. Treating while the tumor remains in place may expose the immune system to a broader range of tumor antigens and provide treated tissue for analysis, but this explanation has not been proved in HCC. This review separates established clinical findings from biological hypotheses, explains how patients could be selected before and after surgery, and proposes a stepwise path for developing perioperative regimens without compromising curative surgery.

## 1. Introduction

Curative-intent resection and local ablation remain central treatment options for selected patients with early-stage hepatocellular carcinoma (HCC), yet local control does not eliminate the risk of recurrence. Recurrence may reflect occult dissemination from the index tumor, genetically independent carcinogenesis within the chronically injured liver, or both [1,2,3,4]. This dual biology complicates systemic prevention: a finite perioperative regimen is most plausibly directed against clonally related residual disease, whereas prevention of de novo tumors requires durable control of viral, metabolic, or other liver injury [5,6,7,8,9,10]. Accordingly, recurrence after curative therapy is not a single endpoint generated by a single biological process.

Recent randomized evidence has clarified the clinical implications. Postoperative sorafenib did not improve recurrence-free survival (RFS) in STORM [11]. The early RFS advantage with atezolizumab plus bevacizumab in IMbrave050 was not sustained with longer follow-up [12,13]. Pembrolizumab did not improve RFS in KEYNOTE-937 [14]. However, postoperative sintilimab improved RFS in a phase II population selected for microvascular invasion [15], and CARES-009 showed that perioperative camrelizumab plus rivoceranib improved event-free survival compared to surgery alone [16]. These findings indicate that systemic therapy is not uniformly ineffective after curative-intent treatment. Instead, outcomes appear to depend on the interaction among treatment timing, regimen, recurrence-risk enrichment, procedure type, liver-disease etiology, and endpoint definition [17,18].

This review evaluates the transition from broadly applied postoperative therapy toward biologically informed perioperative treatment in resectable HCC. It is organized around three unresolved questions. First, what evidence supports the primary tumor and tumor-draining lymphatic network as a clinically relevant antigen source? Second, how should clinical, pathologic, and molecular variables be combined to select patients before and after surgery? Third, what sequence of translational, phase I, randomized phase II, and phase III studies could identify an active regimen while protecting the curative pathway? We compare pivotal and ongoing trials, examine reasons for divergent results, and propose designs that separate the effects of timing, treatment components, and biological selection. The objective is to define the evidence required for durable and generalizable benefit, not to infer a class effect from one positive perioperative trial (Figure 1).

Clinical conclusions in this review are anchored to completed randomized HCC trials. Prospective HCC window and perioperative studies are used to evaluate feasibility, tissue response, and pharmacodynamic signals. Randomized evidence from other cancers is used only to illustrate a timing principle, and preclinical studies are used to define testable mechanisms. Conference abstracts and trial-in-progress reports are treated as provisional and are not interpreted as equivalent to peer-reviewed outcome reports. Because the major trials differ in population, procedure, regimen, time origin, and endpoint, their hazard ratios are described rather than pooled. Throughout the review, biological plausibility, prognostic association, treatment prediction, and demonstrated clinical utility are considered separate levels of evidence.

## 2. Biology of Postoperative Recurrence in HCC

Postoperative recurrence can be conceptualized along two intersecting axes. The first is clonal origin: an event may derive from occult intrahepatic or extrahepatic descendants of the index HCC or represent a genetically independent primary arising from the chronically injured liver. The second is ecological selection: regenerative, vascular, inflammatory, and immune changes after local therapy can suppress or favor either reservoir. The conventional 2-year boundary remains clinically useful, because early events are enriched for tumor-related factors and late events for the carcinogenic field [1,2]. However, elapsed time is an imperfect surrogate for lineage. Genomic comparison of paired tumors found both clonally related and independent recurrences, and the PLANet cohort further identified polyclonal seeding among many early intrahepatic events while distant metastases were usually monoclonal [3,4]. Accordingly, early and late recurrence enrich for, but do not uniquely identify, metastatic and de novo disease, respectively.

Microvascular invasion (MVI), satellite nodules, multifocality, poor differentiation, larger tumor size, and elevated alpha-fetoprotein (AFP) levels each indicate a higher likelihood that dissemination occurred before surgery. MVI is especially informative because it documents vascular entry, but binary scoring discards its extent and distance from the tumor [19]. In a contemporary Western resection cohort, MVI and/or satellitosis identified aggressive recurrence beyond Milan criteria, often eliminating a second curative opportunity [20]. Nevertheless, MVI remains a proxy rather than a direct measurement of residual tumor burden. Perioperative liquid-biopsy studies support a residual compartment invisible to imaging: postoperative circulating tumor-cell burden correlated with recurrence and extrahepatic metastasis, and personalized ctDNA detected molecular residual disease (MRD) even among clinically lower-risk patients [21,22,23].

Hepatectomy also creates a transient postoperative niche. Hemostasis, ischemia-reperfusion, liver regeneration, and wound healing are linked to vascular endothelial growth factor (VEGF) signaling, endothelial activation, and myeloid inflammation. VEGF signaling is required for early regeneration in experimental partial hepatectomy, while cross-tumor models show that surgical inflammation or neutrophil extracellular traps can release dormant deposits from immune control or promote metastatic growth [24,25,26]. These data establish plausibility but do not demonstrate that surgery itself causes recurrence in human HCC. More durable risk resides in the remaining liver: cirrhotic tissue contains expanding mutant clones, chronic injury selects regenerative fitness, and hepatitis B virus (HBV) integration introduces direct genomic disruption [5,6,7]. Steatohepatitis-associated immune suppression and dysfunctional CD8-positive states further illustrate that etiologies are not immunologically equivalent [8,9]. Antiviral therapy can reduce late recurrence in HBV-related HCC [10]. A finite perioperative antitumor regimen is therefore best positioned to target clonally related MRD, whereas prevention of lifelong de novo carcinogenesis requires sustained management of the underlying liver disease.

Key Points: Recurrence Biology

- Recurrence after curative-intent treatment includes both clonally related molecular residual disease and genetically independent tumors arising in the chronically injured liver.
- Early and late recurrence are enriched for these processes, respectively, but recurrence timing alone does not establish clonal origin.
- Finite perioperative systemic therapy is most likely to target molecular residual disease, whereas long-term prevention also requires sustained control of the underlying liver disease.

## 3. The Primary Tumor as an Antigen Source and the Limits of Current Evidence

### 3.1. What the Antigen Source Hypothesis Means

The primary tumor is more than a reservoir of antigen. Viable tumor cells release a heterogeneous set of antigens; dendritic cells acquire and present these antigens; lymphatic drainage connects the tumor to regional nodes; and activated tumor-reactive lymphocytes recirculate through blood and tissue. Programmed cell death protein 1 (PD-1)/programmed death-ligand 1 (PD-L1) blockade can amplify this cycle but does not create antigen specificity by itself. Resection removes the dominant macroscopic reservoir and interrupts continuous drainage from the primary site. It does not eliminate all antigenic stimulation, because occult deposits, antigen-bearing cells, and memory or effector lymphocytes can remain after surgery. The defensible hypothesis is therefore that preoperative therapy may act during a period of greater antigenic diversity and intact lymphatic organization, not that postoperative checkpoint inhibition is biologically impossible [27,28,29,30,31,32,33].

Antigen abundance is not equivalent to effective immunity. Large or biologically aggressive HCCs can contain beta-catenin-associated immune exclusion, dysfunctional lymphocytes, suppressive myeloid populations, hypoxia, and abnormal vasculature [31,34,35,36]. The intact tumor may provide cognate antigen while simultaneously preventing presentation, trafficking, or effector function. Consistent with this tension, clinical activity has been associated with the magnitude of T-cell reinvigoration relative to tumor burden rather than with tumor burden alone [31]. A useful test of the hypothesis must therefore measure effective presentation and immune expansion, not simply the presence or size of the primary tumor.

### 3.2. Evidence Supporting Biological Plausibility

The mechanistic evidence has several levels. In matched mouse models, checkpoint-based therapy initiated before surgery generated stronger systemic tumor-specific immunity and better control of disseminated disease than the same treatment initiated after surgery [27]. Separate experiments showed that tumor-draining lymph nodes contain PD-1/PD-L1-regulated immune populations and contribute materially to checkpoint efficacy [28,29]. Single-cell analyses in human cancers showed that treatment can recruit newly detected tumor-specific clonotypes rather than only reinvigorating lymphocytes already resident in the tumor [30]. Together, these studies explain how an intact tumor-draining-node axis could influence a systemic response, but they do not establish its clinical importance in HCC.

Randomized melanoma trials provide human evidence that moving part of checkpoint treatment before surgery can improve event-free survival [37,38]. The result strengthens the general timing hypothesis because the drug and treatment strategy were compared within the same disease, but the effect size cannot be transferred to HCC. HCC-specific window studies of nivolumab with or without ipilimumab and of cemiplimab demonstrate that brief preoperative exposure can produce major pathologic regression and measurable immune changes [39,40]. Multimodal analysis of neoadjuvant nivolumab likewise identified molecular and pathologic features associated with response [41]. These HCC studies establish pharmacodynamic activity in an intact tumor; their small size and nondefinitive comparators prevent causal conclusions about recurrence or survival.

### 3.3. Evidence Needed to Demonstrate a Clinical Timing Effect in HCC

No completed randomized HCC trial has compared the same regimen initiated before versus after resection while holding the target population, planned total exposure, postoperative treatment, and time origin constant. CARES-009 compared a complete strategy of neoadjuvant camrelizumab plus rivoceranib, surgery, and postoperative continuation with upfront surgery [16]. It therefore establishes efficacy for that integrated strategy. It cannot determine whether benefit arose from the preoperative interval, VEGFR2 inhibition, postoperative continuation, patient selection, or interactions among these elements. The neutral postoperative trials do not fill this gap because they evaluated different drugs, procedures, risk definitions, and treatment clocks.

A direct test should randomize before systemic therapy and use a matched regimen in the preoperative and postoperative groups. Baseline tumor, serial blood, the resection specimen, and standardized postoperative molecular residual disease (MRD) samples should be collected. Prespecified mechanistic endpoints could include expansion and persistence of tumor-reactive lymphocyte clonotypes, dendritic-cell activation, spatial movement of effector cells, change in viable tumor, and clearance of circulating tumor-derived material. These measurements would make the antigen-source hypothesis falsifiable. Even a coherent pharmacodynamic signal would remain supportive rather than definitive unless the preoperative strategy also improved intention-to-treat event-free survival (EFS) without reducing the probability or safety of resection [17].

## 4. Immunologic and Vascular Rationale for Neoadjuvant Checkpoint and Antiangiogenic Therapy

### 4.1. Rationale for Combining Checkpoint and Angiogenesis Inhibition

In HCC, abnormal angiogenesis and immune suppression form a coupled system. Tortuous, permeable vessels promote hypoxia, high interstitial pressure, and poor lymphocyte access, while ineffective immunity permits the abnormal vasculature to persist. The vascular-normalization model proposes that an appropriately dosed angiogenesis inhibitor prunes immature vessels, stabilizes the remaining vasculature, and transiently improves perfusion rather than maximally devascularizing the tumor [42]. Human resection specimens identify heterogeneous immunovascular states, with greater angiogenic activity often accompanying lower immune infiltration [36]. This heterogeneity argues against assuming that VEGF-pathway blockade will convert every immune-excluded HCC into an immune-responsive tumor.

VEGF-A also has direct immunologic effects. It can impair dendritic-cell maturation, promote regulatory T-cell expansion and inhibitory-receptor expression, and create an endothelial barrier that preferentially excludes effector cells [43,44,45,46]. In experimental systems, vascular-normalizing doses improved immune-cell entry. Activated type 1 helper T cells and normalized vessels then reinforced one another, and combined angiogenesis and PD-L1 blockade generated high-endothelial-venule-like structures [47,48,49]. In orthotopic HCC models, dual PD-1 and VEGFR2 blockade increased activated CD8-positive cells, reduced regulatory and suppressive myeloid populations, improved vascular architecture, and outperformed either component alone [50]. These data support a dose-dependent mechanism but do not identify the optimal human perioperative dose.

### 4.2. Human Translational and Clinical Evidence

The preoperative interval could align a transient vascular-normalization window with an intact tumor-draining-node axis. VEGF-pathway inhibition may improve immune-cell access while checkpoint blockade expands tumor-reactive clones, and disseminated cells can be exposed to treatment before postoperative recovery. The resection specimen can then assess viable tumor, perfusion, hypoxia, dendritic cells, tertiary lymphoid structures, and lymphocyte clonotypes. Human studies support individual links: paired biopsies in renal-cell carcinoma showed increased antigen-specific T-cell migration with atezolizumab plus bevacizumab [51], and advanced HCC studies associated benefit from the combination with T-effector and CD8 signatures as well as VEGF-linked regulatory and myeloid resistance programs [52,53]. Applying these findings to microscopic residual HCC remains inferential.

Resectable-disease studies provide direct tissue evidence but limited component attribution. Cabozantinib plus nivolumab enabled margin-negative resection in 12 of 15 patients with borderline or locally advanced tumors and produced deep regression in five; perioperative camrelizumab plus rivoceranib also produced major responses in a small single-arm cohort [54,55]. Responses with checkpoint-only regimens show that pathologic regression is not specific evidence of vascular normalization [39,40]. CARES-009 supplies randomized efficacy evidence for an integrated PD-1 and VEGFR2 strategy, but it did not include monotherapy or postoperative-only combination arms [16]. CAPT poses a more direct timing question by comparing perioperative with postoperative camrelizumab plus rivoceranib, although mature results were not available at the evidence cutoff [56].

### 4.3. Perioperative Agent Dose and Washout Development

Bevacizumab, rivoceranib, lenvatinib, and cabozantinib differ in target location, kinase breadth, reversibility, half-life, hepatic toxicity, and implications for bleeding and wound healing. The dose used for metastatic disease should not automatically be adopted for a short preoperative window. Early development should establish four linked parameters for each regimen: evidence of perfusion or immune trafficking at the selected dose; hepatic tolerability in the intended surgical population; a washout interval compatible with hemostasis and wound healing; and explicit criteria for postoperative resumption. Checkpoint-related hepatitis, endocrinopathy, and other toxicities also require rapid evaluation because even infrequent events can delay an otherwise curative operation.

A practical sequence is to begin with a short window study that reports every enrolled patient’s dose intensity, discontinuation, time to surgery, and reason for non-resection. Small randomized component cohorts can then compare checkpoint blockade alone with the combination and determine whether adding angiogenesis inhibition changes perfusion, immune-cell localization, viable tumor, and perioperative safety. Advancement should require both a coherent pharmacodynamic signal and acceptable surgery completion. A high pathologic response rate among resected patients cannot compensate for preoperative deaths, hepatic decompensation, or loss of operability [17].

Key Points: Biological Rationale and Evidence

- The intact primary tumor and tumor-draining lymphatic network provide a plausible setting for antigen presentation and immune expansion, but no randomized HCC trial has proved that preserving this system improves clinical outcomes.
- VEGF/VEGFR inhibition may improve perfusion and immune-cell trafficking within an agent- and dose-dependent window, while excessive inhibition may increase hepatic, bleeding, and wound-healing risks.
- Perioperative development should establish the contribution, dose, washout, and surgical safety of each component before a regimen advances to definitive testing.

## 5. Critical Comparison of Pivotal and Ongoing Trials

The randomized studies answer different causal questions. STORM, IMbrave050, and KEYNOTE-937 randomized only after local treatment and radiographic clearance; CARES-009 randomized before surgery and evaluated an entire policy comprising neoadjuvant drugs, resection, and postoperative therapy (Table 1). Their time origins, event definitions, procedure mix, comparators, and opportunities for salvage differ substantially. Accordingly, hazard ratios should not be compared as if these trials estimated the same treatment effect. Contemporary consensus emphasizes aligning target population, time zero, estimand, and control arm when early-stage HCC trials are compared [17].

### 5.1. STORM: A Definitive Negative Trial of Postoperative Sorafenib

STORM randomized 1114 patients after resection (81%) or ablation (19%) to sorafenib or placebo. Median RFS was 33.3 versus 33.7 months (hazard ratio [HR], 0.94; 95% confidence interval [CI], 0.78–1.13). Exposure was lower than the intended four years: median treatment duration was 12.5 months, mean daily dose was approximately 578 mg rather than 800 mg, and 89% required dose modification [11]. The translational analysis identified prognostic molecular features but no reproducible subgroup with sorafenib benefit [57]. The prematurely closed SORAMIC ablation cohort was also neutral [59]. These trials exclude a clinically meaningful average benefit from postoperative sorafenib as delivered. They did not test checkpoint inhibition or a perioperative vascular-normalization strategy.

### 5.2. IMbrave050: An Early RFS Signal Not Sustained with Longer Follow-Up

IMbrave050 randomized 668 high-risk patients after resection (88%) or ablation (12%) to one year of atezolizumab plus bevacizumab or surveillance. At 17.4 months’ median follow-up, the prespecified interim analysis met its RFS boundary (HR, 0.72; adjusted 95% CI, 0.53–0.98), with a 12-month RFS of 78% versus 65% [12]. At 35.1 months, median RFS was 33.2 versus 36.0 months and the HR attenuated to 0.90 (95% CI, 0.72–1.12). Overall survival (OS) remained immature (HR, 1.26), and 30% of surveillance patients received atezolizumab plus bevacizumab after recurrence [13]. The RFS curves separated early and subsequently converged and crossed, demonstrating a time-varying treatment effect. This pattern is compatible with temporary suppression or delay of some recurrences, but the curves alone cannot establish whether recurrence was delayed, biologically altered, or redistributed over time.

### 5.3. KEYNOTE-937 and Sintilimab: Contrasting Results with PD-1 Monotherapy

KEYNOTE-937 randomized 959 patients after resection (88%) or ablation (12%) to one year of pembrolizumab or placebo. At 50.7 months’ median follow-up, median RFS was 46.7 versus 45.5 months (HR, 1.06; 95% CI, 0.88–1.26; *p* = 0.719). Descriptive OS was also neutral (HR, 1.08). Grade 3/4 treatment-related adverse events (TRAEs) occurred in 14% versus 5%, and 10% versus 1% discontinued for toxicity [14]. This was a neutral randomized test of postoperative PD-1 monotherapy.

The randomized phase II sintilimab study is notable because it enrolled 198 patients after R0 resection with histologically confirmed microvascular invasion, thereby enriching for early, index-tumor-related recurrence. Six months of sintilimab prolonged median RFS from 15.5 to 27.7 months (HR, 0.534; 95% CI, 0.360–0.792), while grade 3/4 treatment-related adverse events occurred in 12.4% of treated patients and OS remained immature [15]. The result differs from KEYNOTE-937 in several respects, including resection-only enrollment, mandatory postoperative MVI, shorter treatment exposure, and a more homogeneous China-based population. These features increased biological and event-risk enrichment but also limit generalizability. Because all participants had MVI, the study could not test a treatment-by-MVI interaction and therefore does not establish MVI as a predictive biomarker for PD-1 blockade. Its open-label phase II design, modest sample size, and immature OS further preclude routine adoption. The trial should therefore be interpreted as evidence supporting a phase III evaluation of pathology-enriched postoperative PD-1 therapy, rather than proof of a universal adjuvant class effect. In addition, because MVI is identified after resection, it can guide postoperative adaptation but cannot serve as a pretreatment selection criterion without a validated preoperative surrogate.

### 5.4. CARES-009: Efficacy, Component Attribution, and Surgical Safety

CARES-009 randomized 294 patients before resection to two cycles of camrelizumab plus rivoceranib followed by surgery and postoperative combination therapy, or upfront surgery. Eligibility included Barcelona Clinic Liver Cancer (BCLC) stage A tumors larger than 5 cm, BCLC B disease, and selected BCLC C disease without Vp4 invasion or extrahepatic spread. Median event-free survival (EFS) was 42.1 versus 19.4 months (HR, 0.59; 95% CI, 0.41–0.85; *p* = 0.004). Postoperative disease-free survival was similar in direction [16]. This provides randomized evidence that the complete perioperative strategy improved disease control in the study population. The trial cannot determine whether benefit arose from preoperative priming, VEGFR2 inhibition, continued postoperative treatment, or their interaction, and OS remained immature. Safety signals were clinically important: 38% experienced grade 3 or worse treatment-related events, two deaths during neoadjuvant therapy were potentially treatment related, and Clavien–Dindo grade 2 or worse postoperative complications occurred in 35% versus 16%. The China-only enrollment (99% Han Chinese; 77% HBV-associated disease) requires external validation of both efficacy and surgical risk [16,59].

Consequently, the findings should be applied primarily to patients resembling the trial population and treated within a multidisciplinary surgical framework. They should not be interpreted as establishing perioperative therapy as the preferred approach for all technically resectable BCLC B/C tumors.

### 5.5. Ongoing Phase III Programs Awaiting Definitive Readout

No efficacy report was available at the evidence cutoff for several major postoperative phase III programs (Table 2). Trial-in-progress abstracts describe CheckMate 9DX (nivolumab vs. placebo) and EMERALD-2 (durvalumab plus bevacizumab, durvalumab plus placebo, or dual placebo) but contain no outcome data [60,61]. EMERALD-2 is particularly informative because its three-arm design can estimate the incremental contribution of VEGF blockade within a single population. Other programs evaluate toripalimab, postoperative camrelizumab plus rivoceranib, duration of sintilimab in MVI-positive disease, penpulimab plus anlotinib, cadonilimab, or tislelizumab plus lenvatinib. Where no trial-specific scientific report exists, peer-reviewed landscape sources provide design context but cannot substitute for outcome data [62,63,64]. The activity of penpulimab plus anlotinib in unresectable HCC is regimen evidence, not an adjuvant result [65]. Current American Association for the Study of Liver Diseases (AASLD) and European Association for the Study of the Liver (EASL) guidance does not endorse routine adjuvant systemic therapy after curative resection or ablation [66,67]. CARES-009 renews interest in perioperative treatment but does not overturn the negative adjuvant evidence. The present evidence base comprises one positive but geographically concentrated perioperative strategy, a positive pathology-enriched phase II signal, and mature negative findings for several applied postoperative approaches [17,18].

## 6. Reasons for Divergent Results

The divergent results of the postoperative and perioperative trials cannot be attributed to treatment timing alone because timing changed together with regimen, population, exposure, geography, procedure, and endpoint definition.

### 6.1. Timing, Regimen, and Exposure

Timing is biologically compelling but has not been isolated in HCC. CARES-009 differs from STORM, IMbrave050, and KEYNOTE-937 not only by starting before surgery, but also by risk profile, regimen, treatment duration, geography, resection-only eligibility, and the use of an EFS clock [11,12,14,16]. CAPT is the closest randomized timing test, yet mature outcomes remain unavailable [56]. Regimens are also non-exchangeable: sorafenib is a broad multikinase inhibitor; pembrolizumab and sintilimab are PD-1 antibodies; atezolizumab plus bevacizumab couples PD-L1 blockade to VEGF-A neutralization; and camrelizumab plus rivoceranib couples PD-1 blockade with oral VEGFR2 inhibition. Target location, kinase breadth, half-life, dose intensity, washout, and hepatic toxicity all influence perioperative feasibility and efficacy. Treatment schedules also differed. IMbrave050 and KEYNOTE-937 planned approximately one year of postoperative therapy, whereas CARES-009 planned two neoadjuvant cycles followed by surgery and up to 15 postoperative cycles, corresponding to approximately 12 months of total perioperative therapy [12,14,16]. The convergence of the IMbrave050 curves raises the possibility that treatment delayed rather than eliminated some recurrence events, but this remains an interpretation of the time-varying treatment effect rather than a demonstrated biological mechanism [13]. 

CARES-009 should not be interpreted as a timing-controlled counterpart to IMbrave050 or KEYNOTE-937. It enrolled patients considered suitable for hepatectomy at Chinese centers, including patients with BCLC A tumors larger than 5 cm, BCLC B disease, and selected BCLC C disease without Vp4 portal-vein invasion or extrahepatic metastasis. The population was 99% Han Chinese and 77% HBV-associated. The trial also evaluated a PD-1–VEGFR2 combination initiated before surgery and continued postoperatively. In contrast, IMbrave050 and KEYNOTE-937 randomized patients after resection or ablation, enrolled different postoperative risk populations, used different regimens and treatment durations, and measured RFS from a postoperative time origin [12,14,16]. Differences in tumor burden, recurrence risk, surgical selection, procedure type, etiology, geography, treatment exposure, and endpoint definition could each contribute to the divergent results. The cross-trial contrast is therefore hypothesis-generating and cannot estimate the causal effect of treatment timing.

### 6.2. Etiology and Recurrence-Risk Definitions

Etiology can influence tumor immunobiology, background-liver carcinogenesis, and treatment tolerance. HBV-related HCC may retain viral antigens and HBV-integration-associated genomic alterations, while ongoing viral replication contributes to late recurrence. Antiviral suppression is therefore a relevant co-intervention rather than a background characteristic. HCV cure, alcohol-associated liver disease, and metabolic dysfunction-associated steatotic liver disease or steatohepatitis create different inflammatory, fibrotic, and immune states. Although NASH models identified dysfunctional PD-1-positive CD8-positive T-cell states and attenuated immunotherapy activity, trial-level meta-analysis and real-world advanced HCC data have not established a clinically actionable viral-versus-nonviral prediction rule [9,69,70]. Etiology is biologically relevant and prognostic, but it is not a validated predictive biomarker for perioperative checkpoint inhibition.

This uncertainty is especially relevant to CARES-009, which was conducted exclusively in China and enrolled a population that was 99% Han Chinese and 77% HBV-associated. HBV-associated disease accounted for approximately 63% of IMbrave050 and 60% of KEYNOTE-937 [12,14,16]. The efficacy and surgical-safety findings from any one trial cannot be assumed to apply to populations with different proportions of metabolic-, alcohol-, HCV-, or HBV-associated disease. Future perioperative trials should stratify by etiology and region; document HBV DNA suppression, HCV cure, metabolic activity, alcohol exposure, cirrhosis, and liver reserve; and prespecify treatment-by-etiology interactions. A predictive claim requires a randomized interaction, not a comparison of outcomes across regions or etiologic subgroups receiving different treatment strategies.

Definitions of ‘high recurrence risk’ encompassed heterogeneous biological profiles. IMbrave050 used postoperative combinations of size, number, vascular invasion, and grade; KEYNOTE-937 included intermediate through very-high risk; CARES-009 admitted selected BCLC B/C disease; and the sintilimab study required MVI [12,14,15,16]. The control groups reveal quite different event burdens, although their estimates are not directly comparable. Lower-risk populations dilute absolute benefit by including more already-cured patients; extremely high-risk populations increase events but may include systemically disseminated or resistant disease. MVI and satellites are strong prognostic enrichment variables, not proven universal predictors of ICI benefit. Pathology, ctDNA, and marker kinetics should therefore refine clinicoradiologic eligibility while treatment interactions remain prespecified hypotheses.

### 6.3. Resection Versus Ablation and Endpoints That Distinguish Delay from Cure

Resection supplies margins, MVI, satellites, grade, and an on-treatment specimen; ablation defines clearance radiographically, leaves necrotic antigen in situ, and mixes local tumor progression with intrahepatic metastatic and de novo recurrence. This is not evidence that ablation is inferior: the final SURF analysis of small HCC showed similar 5-year RFS after surgery and radiofrequency ablation, and ablation can expand tumor-associated antigen-specific T-cell responses [71,72]. It does mean that mixed-procedure trials require stratification, separate estimands, and explicit classification of local versus new intrahepatic failure. MVI-guided therapy is intrinsically resection-specific.

Postoperative RFS begins after successful local therapy. Perioperative EFS begins at randomization and should count progression, death, toxicity, or loss of operability before resection. EFS therefore appropriately captures a neoadjuvant strategy that compromises the curative pathway, but it cannot be numerically compared with postoperative RFS. Nonproportional hazards further complicate interpretation: a Cox HR averages over time and cannot distinguish permanent eradication from suppression during therapy [73]. Prespecified landmark EFS/RFS, restricted mean survival time, recurrence timing and site, mature OS, liver decompensation, and salvageability should accompany the HR. Crossovers and effective post-recurrence therapy must be reported. The clinically meaningful endpoint is durable cure without sacrificing operability, hepatic reserve, or the chance of curative salvage.

### 6.4. Regional Resectability and the Target Population for Perioperative Therapy

Resectability in HCC has both technical and oncologic dimensions. Technical resectability asks whether complete macroscopic tumor removal is feasible with an adequate future liver remnant and acceptable perioperative risk. Oncologic appropriateness asks whether upfront hepatectomy represents a guideline-supported treatment strategy relative to transplantation, ablation, locoregional therapy, or systemic therapy. These concepts are not interchangeable and depend on liver reserve, clinically significant portal hypertension, tumor number and distribution, vascular invasion, institutional expertise, organ availability, and regional treatment frameworks [67].

Western frameworks generally prioritize resection for localized disease with preserved liver function and an adequate future liver remnant, although selected patients with multifocal disease may undergo resection at experienced centers. BCLC C disease with macrovascular invasion is generally directed toward systemic therapy [67]. CNLC-based practice permits multidisciplinary consideration of resection in selected patients with more advanced but liver-confined disease. Nevertheless, the 2024 CNLC guideline does not recommend resection for most stage IIIa disease and limits consideration to carefully selected cases [74]. CARES-009 enrolled CNLC stage Ib–IIIa disease, corresponding to BCLC A tumors larger than 5 cm, BCLC B disease, and selected BCLC C disease without Vp4 involvement or extrahepatic metastasis [16]. Its ‘resectable’ population therefore does not correspond to a uniform international early-stage surgical population.

For this review, the target population for perioperative therapy is defined as patients with nonmetastatic HCC, adequate hepatic reserve and future liver remnant, and a credible baseline plan for curative-intent resection after multidisciplinary evaluation. Future trials should distinguish: (1) conventionally resectable disease for which upfront surgery is widely accepted; (2) technically resectable but oncologically borderline disease, including selected multifocal tumors or limited macrovascular invasion; and (3) initially unresectable disease treated with conversion intent. The third category addresses a distinct conversion-therapy question and should not be pooled with perioperative trials. Trials should prespecify and stratify tumor stage, extent of vascular invasion, liver function, portal hypertension, region, and the clinical rationale for choosing surgery.

Key Points: Interpreting the Clinical Evidence

- STORM, the updated IMbrave050 analysis, and KEYNOTE-937 do not support routine, broadly applied postoperative systemic therapy after curative-intent treatment.
- The MVI-selected sintilimab trial supports further evaluation of risk-enriched postoperative therapy, whereas CARES-009 supports one perioperative combination strategy; neither establishes a universal immune-checkpoint-inhibitor class effect or isolates the contribution of treatment timing.
- Differences in regimen, exposure, recurrence-risk enrichment, etiology, geography, procedure, time origin, and endpoint definition preclude simple comparisons of hazard ratios across trials.

## 7. Pathologic Response and Surgical Safety

### 7.1. What Pathologic Response Can Establish

Neoadjuvant therapy allows the resection specimen to function as a spatial pharmacodynamic assay, but pathologic response among resected tumors and the clinical outcome of entering the full treatment strategy are different estimands. Brief checkpoint exposure produced extensive regression in subsets treated with nivolumab with or without ipilimumab or with cemiplimab [39,40]. Camrelizumab plus rivoceranib, cabozantinib plus nivolumab, neoadjuvant nivolumab, and tislelizumab plus lenvatinib also produced deep responses in selected patients, although eligibility, denominators, and response thresholds varied [41,54,55,68]. Reporting only patients who reached resection can overestimate the probability of benefit when progression, toxicity, or refusal occurs before surgery.

The NeoHCC patient-level analysis included 111 patients; among 104 evaluable specimens, 32% had at least 70% regression and 18% had a pathologic complete response. Major response was associated with longer relapse-free survival (HR, 0.26), and at least 90% regression best separated outcomes [75]. Final PRIME-HCC conference data were consistent: 30 of 33 treated patients underwent surgery, with major response in 33% of 27 evaluable specimens and complete response in 22% [76]. A locoregional-therapy cohort also supported a 90% threshold [77]. These are patient-level prognostic associations. CARES-009 increased both major response and EFS, but used 50% or less viable tumor as its definition and combined neoadjuvant with postoperative treatment [16]. One trial cannot establish that increasing pathologic response will increase EFS or OS across regimens.

Pathologic response currently has three defensible uses. It can confirm that a drug reached and altered the tumor, help rank regimens in early development, and identify postoperative risk within a treated cohort. It is not yet a validated surrogate endpoint. Surrogacy requires trial-level evidence that between-arm improvements in response consistently predict between-arm improvements in EFS or OS across multiple randomized studies. A comparison of responders with nonresponders within one treatment arm is vulnerable to baseline biological differences and post-randomization selection, and it cannot establish surrogacy.

### 7.2. Standardizing Pathologic Assessment

HCC pathology should record continuous residual viable tumor, necrosis, and regression rather than rely only on study-specific dichotomies. Updated pan-tumor recommendations and the PATHdata reproducibility study provide a standardized foundation [78,79]. Liver-specific protocols should map the tumor bed, sample heterogeneous regions densely, score each lesion, document prior locoregional therapy and baseline necrosis, and report MVI, satellites, margins, grade, and vascular thrombus. Central liver-pathology review should be blinded where feasible. Response Evaluation Criteria in Solid Tumors (RECIST) and modified RECIST are complementary but cannot substitute for tissue; radiographic complete response should not justify omitting an otherwise curative operation outside a dedicated trial.

### 7.3. Surgical Safety as a Coequal Outcome

Surgery is part of the treatment effect, not a downstream administrative event. CARES-009 reported two potentially treatment-related preoperative deaths and more grade 2 or worse postoperative complications with perioperative therapy [16]. Future trials should report time to surgery, completion and reasons for non-resection, planned versus delivered hepatectomy, margin-negative resection, blood loss, transfusion, readmission, and 30- and 90-day mortality for every randomized patient. Complications should use the Clavien–Dindo and International Study Group of Liver Surgery definitions for posthepatectomy liver failure and bile leak [80,81,82]. Pathologic response can support mechanism and early regimen selection. It cannot compensate for loss of operability or replace intention-to-treat EFS and mature OS.

## 8. Biological Selection Across the Perioperative Pathway

### 8.1. Pretreatment Eligibility and Risk Enrichment

Biological selection should begin with a clinically coherent target population rather than an unvalidated molecular cutoff. The first gate is a credible plan for curative-intent resection, adequate future liver remnant and hepatic reserve, and acceptable portal-hypertension and operative risk. The second gate is sufficient recurrence risk to justify exposure before surgery, with emphasis on features linked to index-tumor dissemination such as larger size, multifocality, radiological vascular invasion, suspected satellites, and elevated AFP or DCP. The third gate is regimen-specific safety, including bleeding risk, autoimmune disease, transplant candidacy, viral control, and the ability to proceed rapidly to surgery if toxicity or progression occurs. Patients whose disease is only potentially resectable after major tumor regression belong to a conversion-therapy population and should be studied separately.

Pretreatment stage, serum markers, imaging, ctDNA, and biopsy features are prognostic candidates, not established predictors of benefit. A low-risk population may yield too few preventable recurrences, whereas an extremely high-risk population may contain occult disseminated or immune-resistant disease that a brief perioperative regimen cannot eradicate. Baseline biopsy can enable immune, vascular, and genomic analyses, but it should be required only when the study has a prespecified hypothesis and biopsy is safe; otherwise it can narrow enrollment without improving care. Early trials should stratify on the strongest clinical variables and evaluate candidate biomarkers prospectively rather than deny treatment or surgery on the basis of an unvalidated signature [17,19,20,23,34,35].

### 8.2. Circulating Tumor DNA as a Measure of Molecular Residual Disease

Tumor-informed circulating tumor DNA (ctDNA) is conceptually close to MRD because it tracks patient-specific variants after macroscopic tumor removal. In 88 resected patients followed for a median of 80.7 months, postsurgical MRD positivity within seven days independently predicted recurrence (HR, 2.16), including clinically lower-risk strata [23]. A separate tumor-informed series found that serial ctDNA identified recurrence before imaging and earlier than AFP in many patients [83]. A methylation assay in paired samples from 103 resected patients also associated a high postoperative score with increased 2-year recurrence risk (HR, 3.33) [84]. PRIME-HCC added treatment context: postoperative ctDNA clearance was associated with 2-year RFS of 82% versus 29%, and a reduction of at least 50% also correlated with outcome, although these were small exploratory data [76].

Important limitations remain. Early HCC can shed little DNA, postoperative inflammation increases total cell-free DNA, tissue-informed assays require adequate tumor and matched normal material, tumor-naive assays trade personalization for accessibility, and clonal hematopoiesis or technical errors can generate false signals. Sampling time is not standardized. An immediate postoperative draw may reflect surgical perturbation, whereas a later landmark delays treatment decisions. A negative result lowers but does not eliminate risk, and serial testing is more informative than a single sample. Most importantly, prognostic value is not treatment-predictive value. No completed randomized HCC trial has shown that starting, escalating, switching, or withholding perioperative therapy on the basis of ctDNA improves EFS or OS. A randomized protocol of ctDNA-enhanced surveillance addresses surveillance utility, not yet treatment-selection utility [85].

### 8.3. Serum Marker Kinetics and Control of the Background Liver

AFP and DCP, also called PIVKA-II, are inexpensive and rapidly repeatable. Among 1755 resected patients, incomplete postoperative conversion of either marker was independently adverse [86]. AFP half-life studies also associate delayed clearance with early recurrence and worse survival [87,88]. Interpretation must be limited to baseline secretors and account for expected half-life, active hepatitis, liver regeneration, vitamin K status, cholestasis, and anticoagulation. Marker kinetics are best used as a complementary low-cost layer, not a universal binary MRD test. Control of the background liver belongs in the same strategy: nucleos(t)ide therapy after HBV-related resection improves postoperative prognosis and preferentially reduces late recurrence, which is enriched for de novo carcinogenesis [10,89].

### 8.4. Resection Tissue Spatial Biology and Microvascular Invasion

Tissue provides biological context that plasma cannot. The TIMES model integrated the spatial distribution of five markers and was validated across five cohorts, with reported accuracy of 82.2% and specificity of 85.7% for recurrence classification [90]. In neoadjuvant cabozantinib plus nivolumab, spatial transcriptomics linked response to immune-rich, proinflammatory B-cell and fibroblast states, whereas an early recurrence after major response contained an immune-poor region resembling nonresponders [91]. These studies show how spatial organization can identify resistant niches that abundance alone misses. The signatures are not companion diagnostics: platforms, tissue sampling, lesion heterogeneity, etiology, and preanalytic processing require prospective external validation and a prespecified treatment interaction.

Microvascular invasion (MVI) is routinely available after resection, strongly enriches for early aggressive recurrence, and can be graded by extent rather than presence alone [19,20]. The positive MVI-selected sintilimab trial makes MVI an attractive enrichment variable, but it does not prove that MVI predicts benefit from every checkpoint or antiangiogenic regimen [15]. Because MVI is identified after resection and is sensitive to sampling, it cannot by itself select patients for preoperative treatment. It is better suited to postoperative risk revision and stratified randomization.

### 8.5. A Testable Selection Algorithm

A prospective algorithm should update risk at three landmarks. At baseline, clinicoradiological risk and liver reserve determine trial eligibility, while candidate plasma or tissue markers define stratification factors rather than treatment exclusions. During the preoperative window, toxicity, liver function, unequivocal progression, and a protocol-defined maximum delay trigger rapid surgical review; absence of radiographic shrinkage alone should not cancel a feasible curative operation. After resection, continuous viable tumor, MVI extent, satellites, grade, margins, ctDNA, and AFP/DCP kinetics update residual risk. Patients with persistent MRD or viable immune-excluded tumor can enter an escalation or switch randomization, while patients with deep response and repeatedly negative MRD can enter a duration or de-escalation randomization. Negative MRD does not justify reduced surveillance outside a validated trial.

Each proposed marker must pass four steps: analytical validity, reproducible prognostic validity, a randomized treatment-by-marker interaction, and evidence that acting on the result improves a patient-centered outcome. Assay failure, indeterminate values, conversion between positive and negative states, and missing tissue must be handled in advance. Current markers have reached different points along the first two steps; none has completed the full pathway for perioperative HCC. Biological selection should therefore remain an embedded research strategy rather than a standard treatment rule.

Key Points: Biological Selection

- Pretreatment selection should require both a credible curative surgical plan and sufficient recurrence risk; conversion-intent disease should be studied separately.
- Pathology, ctDNA, AFP/DCP kinetics, and spatial immune features can refine prognosis after resection, but none is a validated treatment-predictive biomarker.
- A biomarker-guided rule should not enter practice until the assay is standardized, a treatment interaction is reproduced, and randomized evidence shows that acting on the result improves outcomes.

## 9. Treatment Development and Trial Strategy

### 9.1. Use a Stage-Gated Development Program

Perioperative treatment should advance through linked stages rather than move directly from metastatic activity to a definitive surgical trial. The first stage nominates an agent or combination using HCC-specific mechanism, activity, hepatic tolerability, and a credible perioperative dose. The second stage is a short window study that establishes feasibility, dose delivery, washout, time to surgery, resection completion, and tissue pharmacodynamics in every enrolled patient. The third stage is a randomized proof-of-concept study that separates the preoperative contribution, the postoperative contribution, or the contribution of angiogenesis inhibition. Only then should a globally representative phase III trial test durable clinical benefit. A negative or unsafe result at one stage should prompt modification or discontinuation rather than automatic progression to the next phase.

Go or no-go criteria should be protocol-specific and declared before enrollment. At minimum, they should integrate surgery completion, treatment-related delay, hepatic decompensation, serious postoperative morbidity, and a pharmacodynamic or clinical efficacy signal. Pathologic response can support early ranking, but it should not be the sole gate, because a regimen can generate regression in resected tumors while harming patients who never reach surgery.

### 9.2. Randomize Before Treatment and Decompose Timing

A definitive timing study should be resection-specific and randomize before systemic therapy. The most informative design would compare immediate surgery followed by a full postoperative course, a short neoadjuvant course followed by surgery without routine continuation, and the same neoadjuvant course followed by postoperative completion. The postoperative portion of the third arm can be shortened so that its total planned exposure matches the first arm; the second arm then tests whether a brief preoperative course has durable value without continued treatment. If three arms are infeasible, a sequential design can randomize timing first and repeat randomization after surgery within pathologic-response or MRD strata. A perioperative-versus-surgery comparison alone retains the component-attribution limitation of CARES-009; cross-trial comparison of perioperative and postoperative camrelizumab plus rivoceranib remains indirect [16,17].

### 9.3. Protect the Curative Pathway

The preoperative window should be deliberately short, generally two or three cycles, with agent-specific antiangiogenic washout, explicit portal-hypertension and liver-reserve criteria, rapid multidisciplinary review, and rescue-to-surgery rules. Randomization should not delay the control operation. An independent monitoring committee needs prespecified boundaries for failure to reach resection, hepatic decompensation, bleeding, wound complications, and 90-day mortality. Surgery completion and serious morbidity should be formally compared using standardized pathology and surgical definitions [78,79,80,81,82]. In a population already eligible for cure, recurrence control cannot be interpreted apart from treatment-related loss of operability or hepatic reserve.

### 9.4. Use Two-Stage Risk Classification and Randomized Adaptation

Pretreatment classification should combine tumor stage, size and number, radiological vascular or satellite features, AFP/DCP secretion, liver function, portal hypertension, etiology, and candidate ctDNA or biopsy features. After resection, risk should be updated with continuous viable-tumor percentage, MVI extent, satellites, grade, margins, spatial immune-vascular state, marker clearance, and ctDNA at a standardized landmark. Deep-response, repeatedly MRD-negative patients can enter a de-escalation randomization; patients with viable immune-excluded tumor or persistent MRD can enter a switch or intensification randomization. Automatic continuation despite absent response has an uncertain biological rationale, but any adaptive strategy must be randomized before it becomes standard. Assay failure and changes in MRD status require prespecified handling [23,85,90].

### 9.5. Choose Endpoints That Separate Cure Delay and Harm

The primary endpoint should be intention-to-treat EFS from preoperative randomization, counting progression, death, or treatment-related failure to complete the curative pathway. Key secondary outcomes should include surgery completion, margin-negative resection, serious morbidity, landmark RFS after resection, early versus late and intrahepatic versus extrahepatic recurrence, mature OS, hepatic decompensation, treatment-free time, patient-reported quality of life, and the proportion of recurrences amenable to curative salvage. The statistical plan should anticipate nonproportional hazards by reporting restricted mean EFS, milestone differences, and time-varying or piecewise effects rather than relying only on a Cox HR [17,73]. Follow-up must extend beyond treatment cessation to determine whether curve separation persists.

### 9.6. Design for Biological and Procedural Generalizability

Global enrollment should prospectively stratify HBV, HCV, metabolic, alcohol-related, and other etiologies; antiviral control; region; cirrhosis and albumin-bilirubin grade; tumor burden; vascular invasion; and planned extent of resection. Conventionally resectable, oncologically borderline, and conversion-intent disease should not share a primary estimand. Resection and ablation should not be pooled unless procedure-specific analyses are prespecified and adequately powered. Optional postoperative transarterial chemoembolization, imaging schedules, and salvage algorithms should be standardized because each can alter recurrence and OS. Biospecimens should be sufficient to validate the proposed biomarker with a locked assay and an external cohort, but feasible enough to avoid restricting the trial to a few highly specialized centers.

Key Points: Treatment Development

- Perioperative regimens should move through mechanism, window-of-opportunity, randomized component-separation, and confirmatory stages with prespecified safety and efficacy gates.
- Randomization before treatment and repeat randomization after resection can separate timing, postoperative continuation, and biomarker-guided adaptation within one development program.
- Success requires durable disease control without reducing surgery completion, hepatic reserve, quality of life, or the opportunity for curative salvage.

## 10. Limitations

This article is a focused narrative review rather than a systematic review. Study identification and selection were not governed by a registered protocol, duplicate screening, or formal risk-of-bias assessment. The evidence base is evolving and includes conference abstracts and trial-in-progress reports with less detail than full publications. Cross-trial comparisons remain indirect because populations, procedures, geographic distributions, liver-disease etiologies, treatment exposures, time origins, and endpoint definitions differ. The intact-tumor and vascular-normalization rationales rely partly on preclinical, cross-tumor, or small translational studies and should not be interpreted as proof that neoadjuvant timing causes clinical benefit in HCC. Candidate markers have mostly been evaluated for prognosis rather than treatment interaction or clinical utility. Finally, positive randomized perioperative evidence is dominated by CARES-009, a China-only, HBV-predominant trial, which limits international generalizability. These limitations justify component-separating, biomarker-embedded, and globally stratified trials rather than immediate extrapolation to routine practice.

## 11. Conclusions

The current evidence supports a narrower conclusion than a simple contrast between adjuvant failure and perioperative success. STORM, the mature IMbrave050 analysis, and KEYNOTE-937 do not support routine, broadly applied postoperative systemic therapy as tested. The MVI-selected sintilimab study supports further evaluation of pathology-enriched postoperative treatment, and CARES-009 demonstrates that one perioperative PD-1 and VEGFR2 strategy can improve EFS in a selected China-based resection population. CARES-009 does not establish that the preoperative component was necessary, that preservation of the primary tumor was the decisive mechanism, or that the benefit generalizes across regions, etiologies, and definitions of resectability.

Progress now requires the biological hypothesis, selection strategy, and treatment-development pathway to be tested together. A timing-controlled trial can determine whether treatment in the presence of the primary tumor changes systemic immunity and clinical outcome. A longitudinal selection program can combine baseline recurrence risk with on-treatment tissue response and postoperative MRD without mistaking prognosis for prediction. A stage-gated development program can identify the dose, component, and duration that justify phase III testing while preserving operability. Until these studies mature, perioperative checkpoint and antiangiogenic therapy should be interpreted as a promising but population- and regimen-specific strategy whose goal is durable cure without avoidable surgical or hepatic harm.

## Figures and Tables

**Figure 1:**
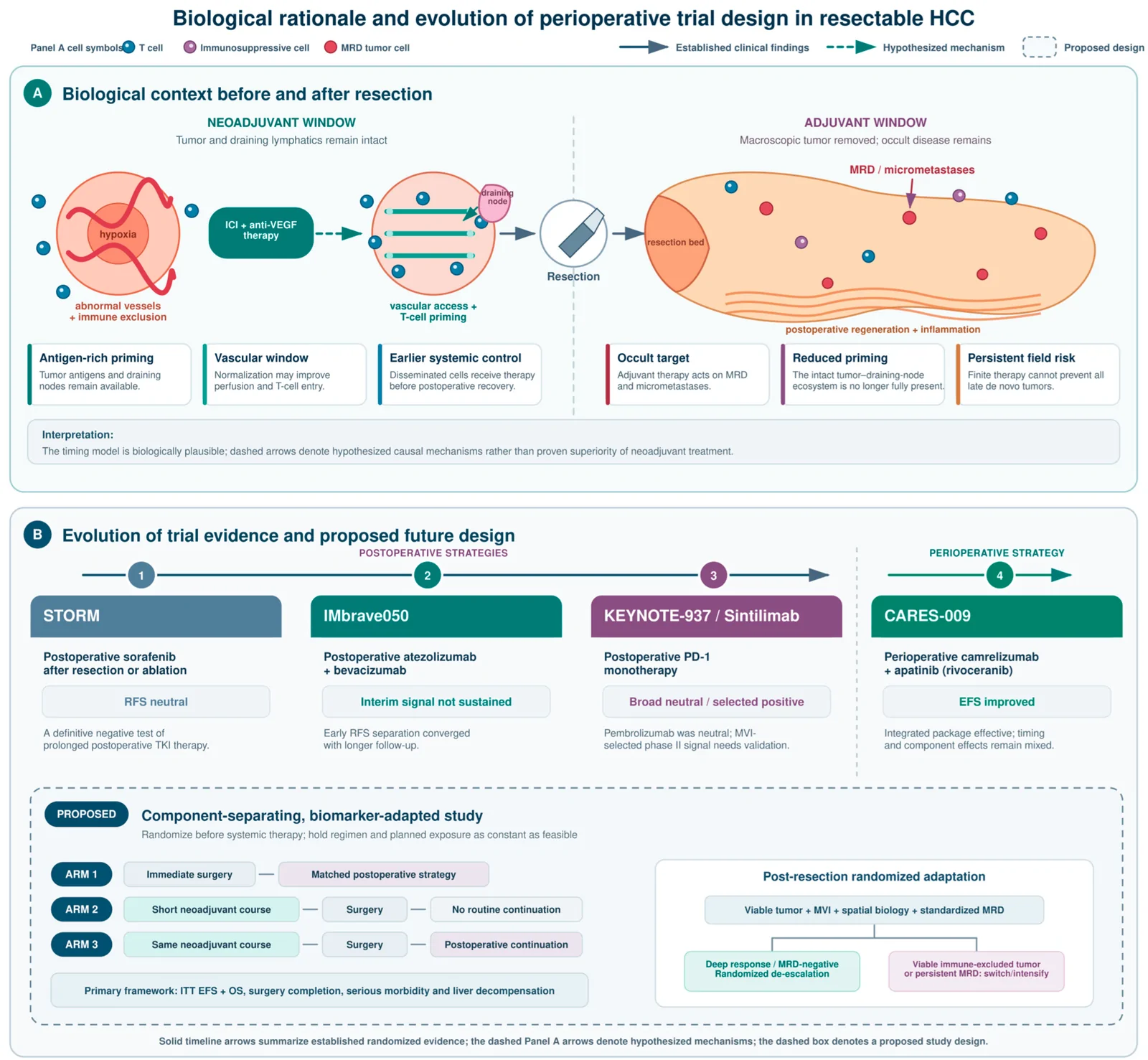
Integrated biological and trial-design framework for perioperative systemic therapy in resectable hepatocellular carcinoma. (**A**) With the primary tumor in situ, immune checkpoint inhibition can act in the presence of tumor antigen and an intact draining lymphatic network, while appropriately dosed VEGF/VEGFR inhibition may transiently improve perfusion and effector-cell trafficking. Preoperative treatment may therefore support tumor-reactive T-cell priming, earlier control of disseminated cells, and an on-treatment tissue readout at resection. After resection, systemic therapy targets molecular residual disease and micrometastases within a milieu shaped by inflammation, regeneration, VEGF signaling, and wound healing, but the intact tumor–draining-node priming ecosystem is no longer fully available and a finite regimen cannot prevent all late de novo tumors arising from the chronically injured liver. Dashed arrows indicate biologically plausible, but unproven, causal mechanisms. (**B**) STORM provided a neutral test of postoperative sorafenib. The interim recurrence-free survival signal in IMbrave050 was not sustained with longer follow-up. KEYNOTE-937 was neutral for postoperative pembrolizumab. The microvascular-invasion-selected phase II sintilimab trial provided a positive pathology-enriched signal requiring phase III validation. CARES-009 demonstrated improved event-free survival with perioperative camrelizumab plus apatinib (rivoceranib), although the integrated design could not isolate the contributions of preoperative treatment, postoperative continuation, VEGFR2 inhibition, or their interactions. Solid timeline arrows summarize established randomized evidence. The proposed future design randomizes before systemic therapy, separates the effects of preoperative timing and postoperative continuation, protects the curative pathway, and incorporates post-resection pathology- and molecular-residual-disease-informed randomized adaptation. The dashed outline denotes a proposed trial design rather than established evidence. Abbreviations: EFS, event-free survival; HCC, hepatocellular carcinoma; ICI, immune checkpoint inhibitor; ITT, intention-to-treat; MRD, molecular residual disease; MVI, microvascular invasion; OS, overall survival; PD-1, programmed cell death protein 1; RFS, recurrence-free survival; TKI, tyrosine kinase inhibitor; VEGF, vascular endothelial growth factor; VEGFR, vascular endothelial growth factor receptor.

**Table 1:** Randomized trials defining the transition from postoperative to perioperative systemic therapy in resectable hepatocellular carcinoma.

Trial	Population/Design	Regimen	Outcomes	Critical Reading	References
STORM (NCT00692770) Adjuvant, phase III	N = 1114; global, double-blind; resection 81%, ablation 19%; protocol-defined high risk 46%.	Sorafenib vs. placebo (up to 4 yrs)	mRFS 33.3 vs. 33.7 mo; HR 0.940 (95% CI, 0.780–1.134). Median treatment 12.5 vs. 22.2 mo; dose modification 89% vs. 38%	Definitively negative. Limited exposure does not create evidence of hidden benefit. No reproducible predictive subgroup. Did not test perioperative vascular normalization or ICI therapy.	[11,57]
IMbrave050 (NCT04102098) Adjuvant, phase III	N = 668; global; resection 88%, ablation 12%; procedure-specific high-risk criteria; HBV 62–63%; CP A.	Atezolizumab + bevacizumab × 12 mo vs. surveillance	Interim HR for RFS 0.72 (adjusted 95% CI, 0.53–0.98). Updated mRFS 33.2 vs. 36.0 mo; HR 0.90 (0.72–1.12). OS immature. TRAEs 89%; grade 3/4 36%; grade 5 1%.	Early RFS separation not durable; curves converge by ~2 yrs. Updated data do not support routine adjuvant use. Crossover complicates OS.	[12,13]
KEYNOTE-937 (NCT03867084) Adjuvant, phase III	N = 959; global, double-blind; resection 88%, ablation 12%; high-risk 71–74%; HBV 59–60%; MVI 57–58%; BCLC A 83%	Pembrolizumab × 1 yr vs. placebo	mRFS 46.7 vs. 45.5 mo; HR 1.06 (0.88–1.26). OS descriptive HR 1.08 (0.81–1.43). Grade 3/4 TRAEs 14% vs. 5%.	Cleanly neutral PD-1 monotherapy test. No efficacy signal despite added toxicity. Await full publication.	[14]
Sintilimab (ChiCTR2000037655) Adjuvant, phase II	N = 198; China; resection only; all MVI-positive.	Sintilimab × 6 mo vs. surveillance	mRFS 27.7 vs. 15.5 mo; HR 0.534 (0.360–0.792). OS immature. Grade 3/4 TRAEs 12.4%.	Positive, pathology-enriched signal; requires phase III validation. Not evidence of universal adjuvant benefit.	[15]
CARES-009 (NCT04521153) Perioperative, phase II/III	N = 294; China; resection-intent only; CNLC Ib-IIIa; HBV 77%.	Neoadjuvant camrelizumab + rivoceranib × 2 cycles → surgery → adjuvant continuation (up to 15 cycles) vs. upfront surgery. One postoperative TACE cycle permitted in both groups.	mEFS 42.1 vs. 19.4 mo; HR 0.59 (0.41–0.85). MPR 35.1% vs. 7.5%; pCR 3.4% in the perioperative arm. OS immature. Grade ≥3 TRAEs 38%; two deaths during neoadjuvant therapy.	First randomized phase II/III trial to improve EFS with a perioperative ICI-VEGFR2 package. Cannot isolate neoadjuvant vs. adjuvant contribution. China/HBV-predominant enrollment. Greater grade ≥2 postoperative morbidity. OS pending.	[16,58]

Notes: Primary efficacy estimates are intention-to-treat unless otherwise stated. Pathologic, safety, and surgical denominators follow each report. Cross-trial comparisons are descriptive because populations, recurrence-risk definitions, procedures, time origins, endpoint definitions, pathology requirements, and follow-up differ. NCT identifiers are provided for trial identification and are not bibliographic references. Abbreviations: BCLC, Barcelona Clinic Liver Cancer; CNLC, China Liver Cancer; CP, Child-Pugh; EFS, event-free survival; HBV, hepatitis B virus; HR, hazard ratio; ICI, immune checkpoint inhibitor; MPR, major pathologic response; mEFS, median EFS; mRFS, median RFS; MVI, microvascular invasion; OS, overall survival; pCR, pathologic complete response; RFS, recurrence-free survival; TACE, transarterial chemoembolization; TRAE, treatment-related adverse event; VEGFR2, vascular endothelial growth factor receptor 2.

**Table 2:** Phase III adjuvant programs awaiting definitive readout: design, operational status, and scientific-publication maturity.

Trial	Population/Design	Regimen	Primary Endpoints	Critical Design Question	Status	References
CheckMate 9DX (NCT03383458) Phase III	Actual N = 545; global, randomized, quadruple-masked; high risk after resection/ablation; CP A.	Nivolumab × 1 yr vs. placebo	RFS by BICR (up to 49 mo)	Will postoperative PD-1 monotherapy differ from KEYNOTE 937, or will any divergence reflect population/clock differences rather than drug effect?	Active, not recruiting. No outcome report yet.	[60,62,63,64]
EMERALD-2 (NCT03847428) Phase III	Actual N = 908; global, randomized, quadruple-masked; high risk after resection/ablation; CP A.	Durvalumab + bevacizumab vs. durvalumab + placebo vs. dual placebo	RFS by BICR (up to 49 mo)	Can VEGF blockade add durable benefit in the postoperative setting and avoid the late RFS convergence seen in IMbrave050?	Active, not recruiting. No outcome report yet.	[61,62,63,64]
JUPITER-04 (NCT03859128) Phase II/III	Actual N = 427; China, randomized, quadruple-masked; resection-only; high risk; CP A.	Toripalimab vs. placebo	RFS by BICR	Will a resection-only, HBV-enriched PD-1 trial behave differently from global mixed-procedure studies, and will results generalize beyond viral HCC?	Active, not recruiting. No scientific readout yet.	[62,63,64]
SHR-1210-III-325 (NCT04639180) Phase III	Actual N = 687; China, randomized; high-risk after resection or RFA/MWA; postoperative TACE allowed.	Camrelizumab + rivoceranib vs. surveillance	RFS by BICR (≈43 mo)	Provides an indirect timing comparison with CARES-009 but cannot isolate neoadjuvant contribution; procedure/TACE heterogeneity may blur interpretation.	Active, not recruiting. No outcome report yet.	[16,62,63,64]
Adjuvant-01 (NCT06089369) Phase III	Planned N = 360; China, randomized; resection-only; MVI-positive; CP A/B7.	Sintilimab × 6 mo vs. 12 mo vs surveillance	RFS (up to 60 mo)	Can pathology enrichment reproduce the phase II signal, and does 12 mo meaningfully outperform 6 mo?	Listed not yet recruiting. No phase III readout. Registry updates lagging.	[15,62,63,64]
Penpulimab-anlotinib (NCT05862337) Phase III	Planned N = 480; China, randomized, quadruple-masked; high-risk after resection or RFA/MWA.	Penpulimab + anlotinib vs. matched placebos	RFS at 3 yrs	Does activity in advanced HCC translate to MRD eradication with acceptable anti-VEGF toxicity in disease-free patients?	Listed recruiting. No outcome report yet.	[62,63,64,65]
COMPASSION-22/AK104-306 (NCT05489289) Phase III	Target N = 570; China, randomized, quadruple-masked; high-risk after resection/ablation; CP A.	Cadonilimab (PD-1/CTLA-4 bispecific) × 16 cycles vs. placebo	RFS by BICR (up to 48 mo)	Can dual-checkpoint activation overcome the antigen-poor postoperative state without unacceptable toxicity?	Enrollment completed. No scientific readout yet.	[62,63,64]
PREVENT-2 (NCT05910970) Phase III	Planned N = 200; China, nonrandomized; resection-only; high-risk; CP A/B7.	Tislelizumab + lenvatinib vs. tislelizumab, × 6 mo	RFS at 3 yrs	Nonrandomized design limits causal inference; tests lenvatinib increment but not absolute value of postoperative PD-1 therapy.	Status unclear. No scientific report yet.	[62,63,64,68]

Notes: Trial identifiers are retained for identification, whereas the reference column cites citable scientific articles or conference abstracts. Trial-in-progress abstracts are not interpreted as outcome evidence. At the revised evidence cutoff, no efficacy readout was identified for CheckMate-9DX or EMERALD-2. References [60,61] are trial-in-progress abstracts without outcome data. [62,63,64] are peer-reviewed landscape analyses. References [15,16,65,68] provide related phase II, perioperative, or advanced-disease regimen evidence and must not be interpreted as results of the listed adjuvant phase III programs. Cross-trial comparisons remain indirect because procedures, recurrence-risk definitions, treatment clocks, etiologies, masking, and endpoint assessment differ. Abbreviations: BICR/BIRC, blinded independent central review/committee; CP, Child–Pugh; CTLA-4, cytotoxic T-lymphocyte-associated protein 4; HBV, hepatitis B virus; MVI, microvascular invasion; MWA, microwave ablation; PD-1, programmed cell death protein 1; RFA, radiofrequency ablation; RFS, recurrence-free survival; TACE, transarterial chemoembolization; VEGF, vascular endothelial growth factor.

## Data Availability

No new data were created or analyzed in this study. Data sharing is not applicable to this article.

## Abbreviations

The following abbreviations are used in this manuscript:

AASLD	American Association for the Study of Liver Diseases
AFP	alpha-fetoprotein
BCLC	Barcelona Clinic Liver Cancer
BICR	blinded independent central review
BIRC	blinded independent review committee
CD8	cluster of differentiation 8
ChiCTR	Chinese Clinical Trial Registry
CI	confidence interval
CNLC	China Liver Cancer staging system
CP	Child-Pugh classification
CRediT	Contributor Roles Taxonomy
ctDNA	circulating tumor DNA
CTLA-4	cytotoxic T-lymphocyte-associated protein 4
DCP	des-gamma-carboxy prothrombin
DNA	deoxyribonucleic acid
EASL	European Association for the Study of the Liver
EFS	event-free survival
HBV	hepatitis B virus
HCC	hepatocellular carcinoma
HCV	hepatitis C virus
HR	hazard ratio
ICI	immune checkpoint inhibitor
MEDLINE	Medical Literature Analysis and Retrieval System Online
mEFS	median event-free survival
mo	month(s)
MPR	major pathologic response
MRD	molecular residual disease
mRECIST	modified Response Evaluation Criteria in Solid Tumors
mRFS	median recurrence-free survival
MVI	microvascular invasion
MWA	microwave ablation
NASH	nonalcoholic steatohepatitis
NCT	ClinicalTrials.gov identifier
OS	overall survival
PATHdata	pathologic response assessment for standardized data collection in neoadjuvant trials
pCR	pathologic complete response
PD-1	programmed cell death protein 1
PD-L1	programmed death-ligand 1
PIVKA-II	protein induced by vitamin K absence or antagonist II
R0	microscopically margin-negative resection
RECIST	Response Evaluation Criteria in Solid Tumors
RFA	radiofrequency ablation
RFS	recurrence-free survival
TACE	transarterial chemoembolization
TIMES	tumor immune microenvironment spatial score
TRAE	treatment-related adverse event
VEGF	vascular endothelial growth factor
VEGF-A	vascular endothelial growth factor A
VEGFR2	vascular endothelial growth factor receptor 2
Vp4	tumor invasion of the main portal vein and/or a contralateral portal vein branch
vs	versus
yr/yrs	year(s)
